# Racism as a Threat to Palestinian Health Equity

**DOI:** 10.1089/heq.2024.0027

**Published:** 2024-06-20

**Authors:** Yara M. Asi, Mienah Z. Sharif, Bram Wispelwey, Nadia N. Abuelezam, A. Kayum Ahmed, Goleen Samari

**Affiliations:** ^1^ School of Global Health Management and Informatics, University of Central Florida, Orlando, Florida, USA.; ^2^ FXB Center for Health and Human Rights, Harvard T.H. Chan School of Public Health, Harvard University, Boston, Massachusetts, USA.; ^3^ Department of Epidemiology, School of Public Health, University of Washington, Seattle, Washington, USA.; ^4^ Division of Global Health Equity, Brigham and Women’s Hospital, Harvard Medical School, Boston, Massachusetts, USA.; ^5^ Boston College William F. Connell School of Nursing, Chestnut Hill, Massachusetts, USA.; ^6^ Mailman School of Public Health, Columbia University, New York, New York, USA.; ^7^ Department of Population and Public Health Sciences, Keck School of Medicine, University of Southern California, Los Angeles, California, USA.

**Keywords:** Palestine, racism, war, health equity

## Abstract

Between October 2023 and April 2024, more than 30,000 Palestinians were killed, and countless others injured, displaced, and traumatized, in the fifth major Israeli assault on the Gaza Strip since 2006. Recent events, along with the trajectory of events over the past 75 years, demonstrate that using a public health framework could help recognize racism as a structural and social determinant of Palestinian health. Using the principles of health equity, we show how Palestinian health inequities are rooted in settler colonialism and racism, amounting to violence and oppression against Palestinian Arabs as a racialized group, regardless of religion or citizenship. Structural racism should be recognized as a driver of Palestinian health inequities.

Between October 2023 and April 2024, more than 30,000 Palestinians were killed, tens of thousands more were injured, and nearly two million were displaced due to the Israeli military campaign initiated after an attack by Hamas in southern Israel. During the first months of the war, around 250 Palestinians were killed each day on average—higher than any major conflict in the 21st century.^1^ More than 70% of the death toll in Gaza is estimated to be women and children.^2^ Concurrently, Israeli forces and settlers have killed more than 400 Palestinians in the West Bank.

The devastation in Gaza, including a collapsed health care system, the destruction of power infrastructure, water sources and storage systems, and sanitation systems, and the killing of health professionals, has drawn widespread media attention and global protests.^3^ Less attention, however, is given to the health inequities, driven by racism and state-sanctioned violence, which Palestinians have been subject to for nearly a century. Anti-Palestinian racism plays a role in Israeli state violence toward Palestinians and in the lack of global action on these and other human rights violations.^4^ While the public health community has otherwise incorporated issues of race, equity, and decolonization in other contexts, there appears to be a hesitation to discuss race and racism in the context of Palestine and Israel. We argue that the routine structural violence required to sustain settler colonialism and unpredictable, ongoing, and racialized population-based direct violence are threats to Palestinian health equity.

Israel’s project of settler colonialism has in effect “rendered settlers indigenous and produces Palestinian natives as alien.”^5^ Many human rights groups, including Human Rights Watch^6^ and Amnesty International,^7^ recognize Israel’s treatment of Palestinians in the occupied territories and within Israel as consistent with the crime of apartheid—a hierarchical system of segregation based on race. Still, this treatment is often justified by Israel and its allies with an unquestionable security rationalization, thereby obfuscating the underlying racialization of Palestinians. Worse, the power imbalances are mislabeled as merely political, religious, or humanitarian issues. Many Palestinian health inequities stem from the othering of Palestinians, regardless of citizenship, as possible security threats, or at the very least, tacit supporters of terrorism and violence. These forms of oppression mirror longstanding global anti-Black racism and the racialization of ethno-religious groups, especially those of Muslim origin,^8^ and serve as the underpinning of excessive rates of discrimination and criminalization.

The racialized Palestinian experience and resulting health outcomes are misrepresented as humanitarian problems with economic and militaristic solutions. Thus, a gap remains in critical analyses of structural and social determinants that undermine the health of Palestinians. Although the etiology of health inequities is complex, we argue that structural racism is undeniably the underpinning of the production, perpetuation, and exacerbation of Palestinian health inequities, and labeling it as such is a vital step in justice and accountability.

## Structural Racism, Violence, and Anti-Palestinian Racism

The structural violence^9^ against Palestinians is rooted in settler and indigenous dynamics that are further complicated by anti-Muslim racism,^8^ serving as mutually reinforcing forms of structural racism. Structural racism is defined as “the totality of ways in which societies foster [racial] discrimination, via mutually reinforcing [inequitable] systems…(e.g., in housing, education, employment, earnings, benefits, credit, media, health care, criminal justice, etc.) that in turn reinforce discriminatory beliefs, values, and distribution of resources”.^10^ As with other forms of racism, a system meant to both “structure opportunity” and “assign value” based on the social construct of race,^11^ this dynamic results in a devaluation of Palestinian life that illuminates widespread Western support for ongoing indiscriminate killing and the withholding of the bare necessities for survival.

Decades of structural violence, racialized discrimination, and human rights violations have stunted Palestinian health care systems across the territories and perpetuated health disparities across populations. The geographical fragmentation of Palestinians has created a hierarchy of Palestinian health outcomes—best among Palestinian citizens of Israel (PCI), worse for Palestinians in the West Bank, and significantly worse for Palestinians in the Gaza Strip. For Palestinians in the occupied territories, life expectancy is nearly a decade shorter than Israelis’, and child, infant, and maternal mortality are several times higher. Regular exposure to violence, dehumanization, humiliation, and hopelessness creates a significant Palestinian mental health burden.^12^ Yet evidence also illustrates discriminatory health access and resulting health disparities for the underserved PCI.^13^ These Palestinians report shorter life expectancies than their Jewish counterparts, higher infant and maternal mortality rates, and greater risk of chronic disease, despite ostensibly having access to the same Israeli health system.^14,15^ Although Palestinian refugees are not included in this discussion of Palestinians living in Israel and the occupied territories, their racialization has prevented many from returning to their homes in Palestine and has cemented their fragmentation.

Of central importance to health equity is the recognition that positioning anti-Muslim racism and antisemitism as opposing forces distracts from our social justice goals and our public health call to prioritize solidarity-building across all racialized and socially marginalized groups. Aside from the reality that Arab Palestinians are of diverse religious backgrounds (Muslim and Christian), Jewish communities have lived for centuries across the Middle East and North Africa, from Morocco to Palestine to Iran. Anti-Muslim racism and antisemitism exemplify how religious minorities are racialized and how racism undermines health in similar ways across ethno-religious groups.^8^

## Health Equity and Anti-Racism in Public Health and Palestine

Guided by Critical Race Theory, public health scholarship has prioritized linking racism to the health of racialized groups for years.^16^ However, discussing racism within mainstream public health organizations became more common in response to the outrage activated by the murder of George Floyd by police in 2020. Several years earlier, physician and epidemiologist Camara Jones outlined three principles of health equity: (1) value all individuals and populations equally, (2) recognize and rectify historical injustices, and (3) provide resources according to need, not equally.^17^ The ongoing violence and myriad injustices for Palestinians provide examples of the critical importance of these principles for health equity and social justice.

In alignment with the first principle, the structural racism embedded in Israeli state policy and practice ensures populations are valued inequitably. The more than 5 million Palestinians living under occupation are controlled by Israeli policies and live under the constant threat of the Israeli military, with no ability to vote in Israeli elections. These Palestinians are subject to movement restrictions at checkpoints and border crossings that pose no barrier to individuals of other nationalities, often leading to the delay or denial of needed health care.^18^ Despite not being Israeli citizens, Palestinians living in the occupied territories are often imprisoned in Israeli jails; of the 4,000 people in administrative detention in Israel from 2006 to 2016, imprisoned indefinitely without charges or a trial, only 35 were not Palestinian. At the end of June 2023, the Israel Prison Service was holding 1,117 Palestinians, including children, in administrative detention, estimated to have doubled since the October 7 attack.^19,20^

Discriminatory policies, such as unannounced military night raids and home demolitions,^21^ directly harm health and are exclusively applied to Palestinians. Palestinians are also at greater risk of environmental racism posed by disproportionate hazards exposure (including lead exposure, water pollution, and inadequate hygiene and sanitation infrastructure).^22,23^ Although Palestinians make up 20% of Israel’s citizens, the 2018 Basic Law codifies Israel as the “nation state of the Jewish people.”^24^ PCI often live in segregated Palestinian towns in Israel with the worst health and socioeconomic indicators, and their children attend under-resourced, segregated schools.^25^

In accordance with Jones’ second principle, the ongoing and historical injustices experienced by Palestinians are rarely recognized or rectified by the Israeli state or multilateral bodies. The realities of settler colonialism and the various forms of violence required to maintain it (including blockade and occupation) are justified, obscured, or even absent in global discourse on Palestine, as well as Israel’s own history books.^26^ Further, Palestinians have been subject to unrecognized historic intergenerational trauma over decades of discriminatory treatment^27^ since the Nakba of 1948, when more than 700,000 Palestinians were forcibly displaced. The racialization of this population has rendered most of them, or their descendants, unable to ever return to their homes in what became the state of Israel, while Israel’s 1950 “Law of Return” allows any Jewish person to immigrate to Israel and attain citizenship.

The cumulative effects of this collective experience, over generations, include poor mental health outcomes for Palestinians.^28^ Yet the global response to Palestine has largely been limited to economic and political initiatives and has neglected to acknowledge, challenge, or provide reparations for the conditions that systematically produce and sustain longstanding inequities. Further, unjust and illegal activities like continued Israeli settlement expansion on Palestinian land are occasionally criticized but never meaningfully countered by global actors.

Lastly, resources are not allocated equally, nor by need, but by race, even within the same geographies. Israel is the only sovereign entity in the area between the Mediterranean Sea and the Jordan River. While the international community continues to recognize Israel as the occupying power of the Palestinian territories, rendering the nation with certain obligations toward Palestinian health and well-being, these obligations remain unfulfilled. Palestinians report significant disparities in terms of health care access, with lower per capita health expenditure ($3,145 in Israel vs. $306 in Palestine in 2017, a 165% difference) and disproportionately fewer physicians, nurses, hospital beds, and facilities for health specialties. In East Jerusalem under Israeli governance, Palestinians pay taxes to the Israeli state but receive few government services.^29^ As Palestinians struggled to procure vaccines for COVID-19, Israel was lauded as a global model of vaccination success.^30^ Palestinians in the West Bank were denied vaccines while Israeli settlers living there were afforded access, despite Israeli public health officials pointing out the pragmatic reality of Israel and Palestine as one epidemiological unit. Israel also engages in active destruction of the Palestinian health system, such as bombing and destroying health infrastructure in Gaza and impeding health access in the West Bank, including blocking ambulances, raiding hospitals, and arresting health workers.^31^

## Declare Palestine a Health Equity Priority

Shortly after the murder of George Floyd, Iyad al-Halaq, a Palestinian student with special needs, was fatally shot by Israeli police on his way to school in Jerusalem. Demonstrations across Israel and the West Bank featured signs likening the two murders: “Justice for Iyad, justice for George,”^32^ building on decades of Black–Palestinian transnational solidarity.^33^ In the US, the attention to Floyd’s murder and the Black Lives Matter movement elevated public recognition of racism as a key driver of health inequities. We were reminded that public health professionals have a moral responsibility to speak out against racism and violence against all socially marginalized populations.

In a moment when Palestinian life is under unprecedented attack, acknowledging the historic and contemporary violence against Palestinians as a form of structural racism that leads to significant health inequities is a necessary step in the call to action for public health professionals to speak up for the Palestinian right to health.^34^ Recognizing structural racism as a health equity threat to Palestinians should be a natural extension of an emerging consciousness about what is needed to dismantle structures of oppression. As public health professionals in pursuit of equity *for all*, our roles require us to advocate for the health and well-being of Palestinians.

## Notes

### Abbreviation Used

PCI: Palestinian citizens of Israel
